# Evolution of Fentanyl Prescription Patterns and Administration Routes in Primary Care in Salamanca, Spain: A Comprehensive Analysis from 2011 to 2022

**DOI:** 10.3390/healthcare12161619

**Published:** 2024-08-14

**Authors:** Cristina Torres-Bueno, Mercedes Sanchez-Barba, Jose-Antonio Miron-Canelo, Veronica Gonzalez-Nunez

**Affiliations:** 1 Faculty of Medicine, University of Salamanca, 37007 Salamanca, Spain; critorresbueno@gmail.com; 2 Department of Statistics, Faculty of Medicine, University of Salamanca, 37007 Salamanca, Spain; mersanbar@usal.es; 3 Institute of Biomedical Research of Salamanca (IBSAL), 37007 Salamanca, Spain; 4 Department of Biomedical and Diagnostic Sciences, Faculty of Medicine, University of Salamanca, 37007 Salamanca, Spain; 5 Instituto de Neurociencias de Castilla y León (INCYL), University of Salamanca, 37007 Salamanca, Spain; 6 Department of Biochemistry and Molecular Biology, Faculty of Medicine, University of Salamanca, 37007 Salamanca, Spain

**Keywords:** opioids, fentanyl, consumption, DHD, transdermal route

## Abstract

(1) Background: The escalating use of opioids contributes to social, health, and economic crises. In Spain, a notable surge in the medical prescription of opioids in recent years has been observed. The aim of this work was to assess the consumption rate of fentanyl, categorised by the different administration routes, in Primary Care in the province of Salamanca (Spain) spanning the years 2011 to 2022, and to compare it with the national trend and with data from the US. (2) Methods: Doses per inhabitant per day (DHD) were calculated, and interannual variations, as well as consumption rates, were subject to thorough analysis. (3) Results: The prevalence of fentanyl use in Salamanca has doubled from 1.21 DHD in 2011 to 2.56 DHD in 2022, with the transdermal system (TD) as the predominant administration route. This upward trajectory mirrors the national trend, yet the rise in fentanyl use is markedly lower than the reported data in the US. This finding may be attributed to an ageing population and potentially inappropriate fentanyl prescriptions, i.e., for the management of chronic non-cancer pain and other off-label prescriptions. (4) Conclusions: The use of fentanyl in Salamanca, particularly through transdermal systems, doubled from 2011 to 2022, aligning with the national trend. Preventive measures are imperative to prevent fentanyl misuse and moderate the observed escalation in consumption rates.

## 1. Introduction

Opioids, well known in healthcare for their pivotal role in pain relief, have been a subject of historical social and political controversy, notably during events such as the “Opium Wars” in the late 19th century. In Spain, until the 1990s, pervasive myths and longstanding fears influenced opioid usage, prompting the issuance of a 1994 ministerial decree to regulate their prescription and foster a more rational approach. Historically, Spain maintained lower opioid use compared to other European countries, but recent years have witnessed a significant surge. A report by the Spanish Agency of Medicines and Health Products (AEMPS) revealed an 80% increase in opioid use from 2008 to 2015 [1]. Notably, Spain transitioned from being the fifteenth country with the highest rates of fentanyl use in 2000 to the fifth position in 2014, potentially attributed to chronic use and off-label prescriptions for non-cancer patients [1].

In 1998, fentanyl, a small molecule that interacts with the μ opioid receptor and that is 50 times more potent than morphine, was approved in Spain. The approval of fentanyl marked a shift in usage patterns: there was an increase in the use of fentanyl and a decrease in the use of morphine, which has previously been associated with the terminal stages of chronic diseases. Fentanyl and its derivatives, remifentanil and sulfentanyl, are used worldwide in intensive care units (ICUs) for the management of pain in critically ill and mechanically ventilated patients [2,3,4]. In paediatric intensive care units (PICUs), fentanyl is the most common opioid for analgosedation, usually in combination with a benzodiazepine (often midazolam) in Europe [5] and Japan [6]; in the latter country, a combination of fentanyl with paracetamol is also the preferred choice for eliciting analgesia in PICUs [6].

Fentanyl is available in a variety of forms with different routes of administration and rates of absorption, ranging from rapid absorption by the sublingual route to prolonged release via transdermal patches. Fentanyl’s various forms led to an increased utilisation, particularly with transdermal patches becoming the predominant mode of administration, followed by oral and nasal forms, whereas injectable formulations are a minority [7]. Currently, there are two indications for the prescription of fentanyl: treating chronic cancer pain with sustained-release transdermal patches in patients on opioid maintenance therapy; and managing breakthrough (irruptive) cancer pain with immediate-release forms (nasal and oral mucosal absorption forms), which pose a higher risk of addiction [8,9,10]. In Spain, the exclusion criteria for prescribing immediate-release fentanyl are non-oncological pain; patients with a history of substance abuse, as they are considered vulnerable to develop a substance use disorder (SUD); and, finally, patients who have not previously been treated with strong opioids [11].

As mentioned above, another important issue with opioid prescription is the risk of addiction. It is well known that opioid misuse is a major social problem in the United States [12], where the rates of addiction to these drugs are higher than in other countries, but it is also present in other countries such as Canada [13]. The evolution of the opioid crisis in the US has taken place in three periods: From 1999 to 2010, characterised by an increase in deaths from prescribed opioids; from 2010 to 2013, when heroin deaths increased, but prescription opioids continued to lead the way; and from 2013 to the present, when deaths from synthetic opioids took centre stage, surpassing death rates from natural opioids [14]. It is at this stage that fentanyl abuse shows high prevalence. At present, fentanyl abuse has become a prevalent concern in the United States, affecting individuals with Opioid Use Disorder (OUD) through both the conscious consumption and inadvertent ingestion of adulterated drugs [15,16,17].

Although Spain does not currently face a problem of the same magnitude, the exponential growth in opioid prescriptions has drawn the attention of health authorities, leading to the publication of an alert by the AEMPS in February 2018 [18]. This alert highlighted concerns, revealing that 40% of primary care prescriptions in 2016 were for non-oncological pain, and that 60% of cases of opioid abuse and dependence informed to the pharmacovigilance system were reported for patients not adhering to fentanyl’s prescribed conditions. Immediate-release fentanyl use for non-oncological pain management reached 40% in 2016 [18]. A retrospective cross-sectional descriptive study conducted between 2014 and 2017 by physicians at the Hospital “12 de Octubre” in Madrid, Spain, found that the main off-label indications for which fentanyl was prescribed were ulcer and wound healing, and chronic non-oncological pain [19]. Over the past decade, fentanyl misuse in Spain has surged, leading to a fivefold increase in treatment admissions for abuse or dependence from 2014 to 2019 (28 in 2014 to 103 in 2019) [20]. Similarly, the number of emergencies related to opioid misuse has significantly increased in recent years: reports indicate that the number of hospital emergencies among users of psychoactive substances informed in 2018 and 2019 is 50% higher than in the previous decade (44 in 2009 and 111 in 2019) [21]. According to the Annual Report of the International Narcotics Control Board (INCB) of the UN in 2023, 13 high-income countries accounted for 78.2% of the global consumption of fentanyl, with Germany (19%), Spain (12.9%) and the United States (12.8%) being the three countries with the highest reported percentages [22]. Other European countries were France, Italy, the Netherlands, Belgium, the United Kingdom, Greece, and Austria.

Opioid use is very common in individuals over 50 years, with a higher prevalence in women: in 2019–2020, the rates were 20.8% in women and 19.7% in men of 55–64 years of age [21]. This age group has the highest prevalence of chronic pathologies associated with pain and is also at greater risk of developing opioid abuse. In the Spanish region of Castilla y León, opioid use doubled between 2000 and 2006, with a threefold increase in costs to the health system [23]. In terms of dosage forms, fentanyl transdermal patches were the most widely used pharmaceutical form in Castilla y León during this period, accounting for between 80% and 90% of the total, while immediate-release forms were in the minority. Given the importance of fentanyl misuse and the lack of current data on fentanyl prescriptions in Castilla y León, we aim to determine the prevalence of fentanyl use in the province of Salamanca, both total consumption and in its main forms of presentation; in addition, this study aims to assess the prevalence of fentanyl use in Salamanca, comparing it with national AEMPS data and available information from the US.

## 2. Materials and Methods

### 2.1. Data Search Using Official Websites

Information from the following official websites was searched and analysed.

- Data on the use of immediate-release fentanyl in Castilla y León were searched for in the Portal del medicamento del Sacyl (Drug Information Centre in Castilla y León) https://www.saludcastillayleon.es/portalmedicamento/es/ (accessed on 8 May 2024) [24].
- Data on fentanyl misuse in Spain were obtained from the Spanish Observatory on Drugs and Addictions (OEDA): https://pnsd.sanidad.gob.es/profesionales/sistemasInformacion/home.htm (accessed on 8 May 2024) [20].
- In terms of the use of opioid medicines in Spain, data on the consumption rates of fentanyl, expressed in DHD, and reports on the prescription of immediate-use fentanyl were searched for in the Observatory on the Use of Medicines, Spanish Agency for Medicines and Health Products (AEMPS) https://www.aemps.gob.es/medicamentos-de-uso-humano/observatorio-de-uso-de-medicamentos/utilizacion-de-medicamentos-opioides-en-espana/ (accessed on 8 May 2024) [25].
- Data on the prevalence of the consumption of fentanyl in the US were found at the National Survey on Drug Use and Health (NSDUH), Substance Abuse and Mental Health Services Administration (SAMHSA) https://www.samhsa.gov/data/data-we-collect/nsduh-national-survey-drug-use-and-health (accessed on 8 May 2024) [26].

### 2.2. Access to Open Public Data

A request for data on fentanyl use in Salamanca was made through the transparency website of the Regional Ministry of Health of the Junta de Castilla y León (https://www.saludcastillayleon.es/transparencia/es?locale=en_ES) (first accessed on 12 January 2022). From the health website, we were directed to the Castilla y León Transparency and Citizen Participation website (https://www.tramitacastillayleon.jcyl.es/web/jcyl/AdministracionElectronica/es/Plantilla100Detalle/1251181050732/_/1284539839313/Tramite) (first accessed on 14 January 2022), where we were referred to the Pharmaceutical Supply Service of the Regional Health Management (GRS) (prestacionfca.grs@saludcastillayleon.es). We were required to submit a comprehensive report detailing the nature and scope of the intended project. Upon the successful submission of our proposal, we promptly received, from CONCYLIA, the requested data regarding the consumption of fentanyl (ATC CODE: N02AB03) from 2011 to 2022 in Primary Care. The data were split by route of administration (oral, nasal, sublingual, and transdermal) and were expressed in terms of the number of patients, containers, and defined daily doses (DDDs) prescribed in Primary Care in the province of Salamanca, Spain. Injectable fentanyl was not analysed, as it is used in inpatient care to treat acute pain (mainly in ICUs) in post-surgery or as an analgesic in general or local anaesthesia, as analgesic premedication for the induction of anaesthesia, and as an adjuvant in the maintenance of general and local anaesthesia.

The Population Register of Salamanca (https://estadistica.jcyl.es/web/es/estadisticas-temas/padron-continuo.html) (accessed on 16 May 2023) was consulted to ascertain the population figures from 2011 to 2022.

This research was conducted exclusively with open public data, adhering to the transparency guidelines outlined by the Transparency website and the Regional Health Administration of Castilla y León. No personal data, as defined by the Spanish General Data Protection Law (LGPD), was utilised for biomedical research purposes, and all data used are entirely anonymous, ensuring there is no possibility of re-identification.

### 2.3. Data Analysis

DHDs (doses per inhabitant per day) were calculated using the following formula:

$$\mathrm{No}.\;\mathrm{DHD}=\frac{\mathrm{No}.\;\mathrm{DDD}\times 1000\;\mathrm{inhabitants}}{\mathrm{No}.\;\mathrm{inhabitants}\left(\mathrm{data}\;\mathrm{from}\;\mathrm{population}\;\mathrm{register}\right)\times 365\;\mathrm{days}}$$

The percentages represented by each route of administration were calculated for each year studied using Excel software (Microsoft Office Professional Plus 2019). Year-to-year variations over the past decade and the consumption rate relative to the average were systematically calculated. Regression analyses were conducted using GraphPad Prism. This analytical tool facilitated the evaluation of statistical differences between slopes, enhancing the robustness of our findings.

## 3. Results

### 3.1. Prevalence of Fentanyl Use in the Province of Salamanca

**Figure 1:**
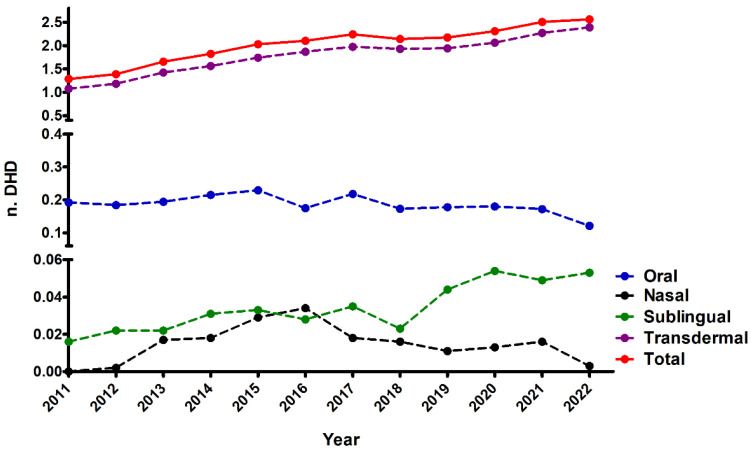
Prevalence of fentanyl use in the Spanish province of Salamanca between 2011 and 2022. DHD data are shown for total fentanyl use as well as by route of administration (transdermal, buccal, nasal, and sublingual).

Utilising data sourced from the Pharmaceutical Supply Service of the Regional Health Administration (CONCYLIA) and the regional population register, we conducted an extensive analysis of the trajectory of fentanyl consumption in Salamanca spanning the years 2011 to 2022 in Primary Care. Defined Daily Doses (DDDs) per 1000 inhabitants per day (DHDs) were calculated for total fentanyl prescriptions, categorically segregated by administration routes: oral, nasal, sublingual, and transdermal. Our findings revealed a consistent and noteworthy upward trend in fentanyl consumption over the study period (Figure 1). DHDs exhibited a twofold increase, rising from 1.21 DHD in 2011 to 2.56 DHD in 2022. Statistical analyses indicated a positive and significant upward trend for total fentanyl use, as well as for the sublingual and transdermal routes of administration, despite the latter being a minority route. Conversely, formulations for immediate-release fentanyl did not display a significant increase in DHDs throughout the study years, showing no statistically significant trend. While transdermal fentanyl mirrored the overall trend in total fentanyl use, as evidenced by equivalent slopes from linear regression analysis (Table 1), the same did not hold true for the sublingual route of administration.

**Figure 2:**
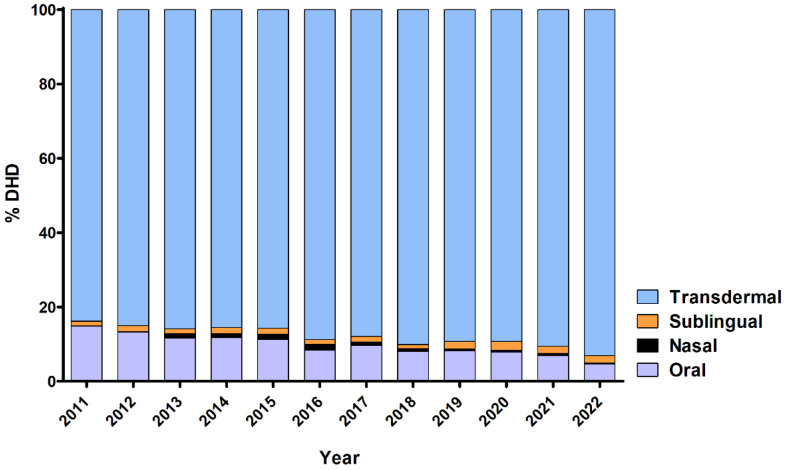
Distribution of fentanyl use by route of administration in the province of Salamanca between 2011 and 2022, expressed as percentage of total consumption. The majority of fentanyl prescriptions are for transdermal use, which also increases over the years to the detriment of the oral route.

**Figure 3:**
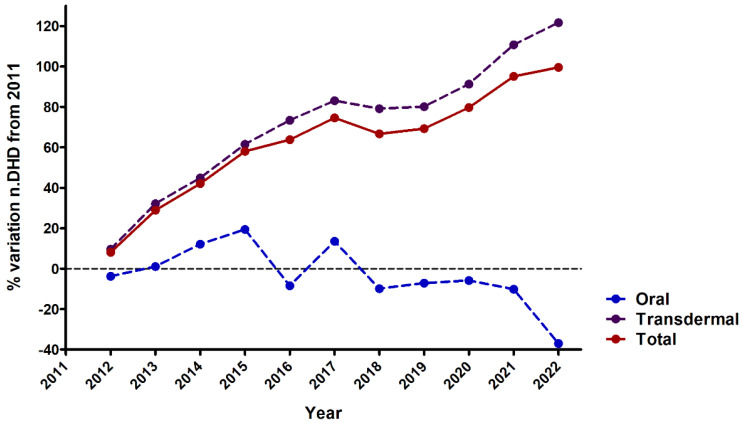
Change in fentanyl use in the province of Salamanca between 2011 and 2022, expressed as percentage of variation in DHDs compared to 2011. Data are calculated for total fentanyl prescriptions, as well as for transdermal and oral formulations. There is a significant increase in the use of fentanyl, mainly due to the transdermal route, while the use of the oral formulation has remained constant.

The predominant mode of fentanyl use was the transdermal route, followed by the oral route (Figure 2), which maintained relative stability over the observed period. A discernible surge in nasal fentanyl use (depicted in black) was noted between 2013 and 2016, followed by a reversal in 2017. From analysing the fold change in DHD compared to 2011 (the initial year of our study), it was observed that the escalation in fentanyl use was primarily attributable to the transdermal route (Figure 3), supported by a positive and statistically significant upward trend in both the transdermal route and total fentanyl use (Table 2). An examination of year-to-year variation indicated a subtle deceleration in the increase in transdermal and total fentanyl consumption between 2013 and 2018, followed by a renewed upward trajectory from 2018 onwards (Figure S1).

### 3.2. Comparison of the Prevalence of Fentanyl Use between Salamanca and Spain

**Figure 4:**
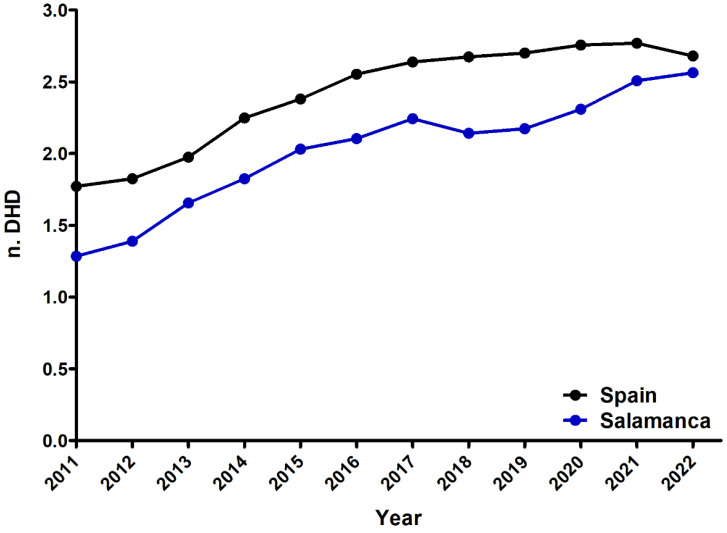
Prevalence of fentanyl use in the Spanish province of Salamanca and in Spain between 2011 and 2022.

We conducted an analysis to ascertain whether the trajectory of fentanyl use in Primary Care in Salamanca aligns with the national trend in Spain. We compared the consumption rates, measured in DHD, between the province of Salamanca and the entire nation (Figure 4). Additionally, we examined the percentage change in DHD relative to 2011 (Figure S2) and the year-on-year variation (Figure S3). Notably, all analyses revealed a comparable increase in both cases, with Salamanca exhibiting a marginally higher year-on-year consumption rise in recent years. These findings indicate a positive and statistically significant upward trend in fentanyl consumption for both Salamanca and the national dataset, with no discernible differences between slopes (Table 3). This suggests that the escalation in DHDs in Salamanca closely mirrors the trend observed for the entirety of Spain. However, it is noteworthy to mention that while the overall pattern is analogous, the initial rate of increase in 2011 is slightly more pronounced in the national data than in the province of Salamanca.

### 3.3. Comparison of the Prevalence of Fentanyl Use between Salamanca and US

Data regarding the prevalence of fentanyl use in the United States were available as a percentage of the total population, whereas no data expressed in doses per inhabitant per day (DHD) was found. The data were sourced from the National Survey on Drug Use and Health (NSDUH) conducted by the Substance Abuse and Mental Health Services Administration (SAMHSA). This information is only accessible from 2015 onwards, as reports from previous years did not disaggregate opioid drugs, with the exception of oxycontin. Moreover, from 2015 to 2021, data are from any use of fentanyl products among population aged 12 or older, expressed in percentages. In 2022, data are estimates of medical fentanyl products and do not include illegally made fentanyl. Consequently, these data are difficult to compare to the reported DHDs in Spain and our evaluation was restricted to assess the consumption rate expressed as patients per 1000 inhabitants between the US and Salamanca from 2015 to 2021, as this variable was not available for Spain.

**Figure 5:**
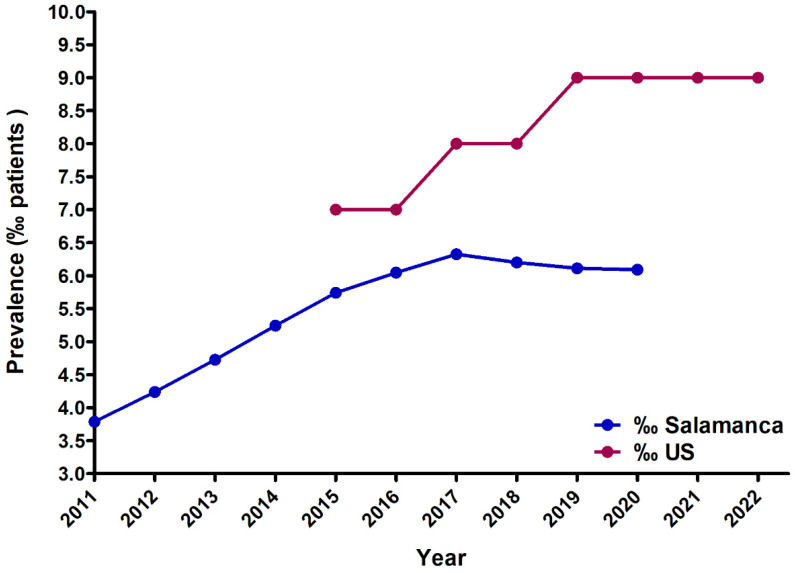
Comparison of the prevalence of fentanyl use in Salamanca and the US, expressed as a percentage (‰) of users in the total population. US data are from the National Survey on Drug Use and Health (NSDUH) of the Substance Abuse and Mental Health Services Administration (SAMHSA). There is an upward trend for total fentanyl use in both Salamanca and in the US.

Our analyses revealed a higher prevalence in the United States compared to our province (Figure 5). In both cases, a positive upward trend was observed and the slope obtained for the US data is steeper. This suggests a more pronounced increase in prevalence over the specified period in the United States relative to Salamanca.

## 4. Discussion

Our findings underscore an undeniable surge in fentanyl usage across developed countries, evident in Salamanca, Spain, and the US, irrespective of the underlying healthcare system. Notably, the escalation in Salamanca is predominantly attributed to the transdermal route of administration, as opposed to immediate-release formulations, which are associated with a higher risk of addiction. In recent years, this upward trend has outpaced the national average in Salamanca, potentially due to the accelerated ageing of the population. The pronounced ageing rate, twice that of the national average, is a predisposing factor for the appearance of chronic diseases wherein pain management poses a significant challenge, leading to a higher demand for primary and hospital care.

However, the relief of chronic non-cancer pain is not an indication listed in fentanyl’s label indications. Furthermore, as noted above, studies show that major opioids like fentanyl are often prescribed for the treatment of non-cancer pain. In this line, a recent study performed with data from the National Health Insurance Claims Database in France has found that the prevalence of off-label prescriptions of transmucosal immediate-release fentanyl (TIRF) was 51.8%, and that 81.7% of the patients did not have a cancer diagnosis [27]. Also, the German Working Group on Tumor Pain has raised some concerns regarding the use of immediate-release fentanyl forms, due to their high risk of a fast development of addiction and tolerance; likewise, this working group has highlighted the need for clear recommendations of use to avoid off-label rapid-onset fentanyl prescriptions [28]. To prevent these situations, it would be advisable to start prescribing less potent opioids, or even non-opioid drugs, according to the WHO analgesic scale, before resorting to a drug with such potency and risk of addiction. An alternative to avoid the development of addiction to immediate-release forms of fentanyl would involve opioid rotation and close monitoring of the patients by a specialised palliative care team [29].

Another of the conditioning factors to be analysed would be the prevailing trends, as it can be observed that the nasal route behaves in peaks or outbreaks, characterised by a transient increase lasting only a couple of years: its use increased between 2013 and 2016, reverting to a minority form of presentation in the subsequent years under study. This prescription pattern may be related to the influence of the pharmaceutical industry, along with the involvement of health visitors in health centres and hospitals.

According to the most recent report of the European Union Drugs Agency (EUDA) in 2024, fentanyl and its derivatives such as carfentanyl have been associated with some episodes of fatal poisoning in Europe [30]. In 2022, 162 overdose deaths were reported for fentanyl in Europe, and it seems that they may be caused by diverted fentanyl for therapeutic use rather than illicit fentanyl [30]. However, these drugs were not the major causes of drug-induced deaths in Europe, except for some outbreaks in some Baltic countries (mainly Estonia) [30]. However, the EU authorities have raised some concerns that these opioids may have a negative impact on public health in EU countries in the near future. In fact, a recent study found fentanyl in wastewater samples from all European countries except for Slovakia; these traces mainly correspond to the pharmaceutical use of fentanyl, but the fact that part of it may derive from illicit fentanyl cannot be ruled out [31]. Nevertheless, the situation in the EU is not as alarming as that of the US, where 6166 overdose deaths were reported in 2021; these differences may rely on the distinct standards of public health and healthcare provision in the EU and US and the high prevalence of illicit fentanyl misuse in the US [32].

Regarding the US, updated open data on fentanyl consumption doses and administration routes are currently unavailable. Therefore, our comparison is based solely on consumption rates. In Salamanca, these are real data on the number of fentanyl prescriptions in Primary Care, while for the US, we only obtained indirect data from consumption surveys. In the case of the US, only data from the National Survey on Drug Use and Health (NSDUH) from the Substance Abuse and Mental Health Services Administration (SAMHSA) were obtained [26]. This survey is analogous to the Survey on Alcohol and Other Drugs in Spain (EDADES report) from the Ministry of Health [20]. The US data encompass any reported use or abuse of fentanyl within the past year among individuals aged 12 years and older since 2015. It is important to note that in prior years, opioid drug data were not disaggregated, except for oxycontin. Consequently, there is a possible underestimation of fentanyl use in the US data, as they are based on reported rather than actual consumption figures. Differences in the healthcare system relative to Spain (universal healthcare in Spain vs. a system largely provided by private companies in the US), together with the influential role of the pharmaceutical industry, could be factors contributing to increased fentanyl consumption in the US.

In the US, prescriptions are issued by hospital doctors, dentists, nurses, and physiotherapists, unlike in Spain, where only registered doctors are authorised to prescribe these medications. In Spain, Primary Care physicians handle the majority of prescriptions, which ensures better monitoring, continuity of care, and home-based interventions at the primary care level. Another possible reason for the higher prevalence of fentanyl and other opioid use in the US, as compared to other countries, may be attributed to cultural factors. The American culture, characterised by greater tolerance towards these drugs, is further influenced by the significant presence of insurance companies and the prominence of private healthcare.

Therefore, the observed results show a persistent upward trajectory of fentanyl use in Salamanca, despite the health alert issued by the AEMPS in 2018. Addressing this worrying trend requires the implementation of new measures. Among them, it would be interesting to update the training of Primary Care professionals, as well as to develop comprehensive clinical guidelines and to implement prevention and public health measures. The aim of this approach is to avoid inappropriate prescribing of both transdermal fentanyl and rapid-release fentanyl for the management of chronic oncological and non-oncological pain.

An important milestone in this direction occurred on 21 July 2021, with the publication by the Ministry of Health of the plan for the optimisation of the use of opioid analgesics for chronic non-oncological pain in the National Health System [33]. This document emphasises that fentanyl prescriptions should be strictly in line with the indications authorised in its Summary of Product Characteristics (SPCs). Also, immediate-release fentanyl medications are subject to health control, requiring the approval of the Medical Inspectorate of each Health Area [33]. However, this study reveals that the increase in fentanyl consumption is not attributed to quick-release forms, but to the prevalent use of transdermal patches. While transdermal patches allow the sustained release of the analgesic drug with less addictive potential, their inclusion in new ministerial orders or opioid programming and optimisation plans in the National Health System is considered necessary. In addition to this, greater pharmacovigilance is crucial to monitor the prescription, use, and possible chronic abuse of transdermal patches. Finally, it would be necessary to raise awareness among patients and their families about the inherent risks of substance use disorder (SUD) associated with chronic opioid use, especially fentanyl due to its high potency. It is imperative to assess each patient’s individual risk of addiction using scales such as the COMM (Current Opioid Misuse Measure) [34] or the SOAPP-R (Screener and Opioid Assessment for Patients with Pain) [35]. Patients subjected to high doses of opioids should be referred to specialised consultations where they can benefit from closer monitoring by healthcare professionals working in interdisciplinary teams [29]. This interdisciplinary approach is essential to address the complex challenges posed by the escalating use of fentanyl [36].

## 5. Conclusions

Fentanyl use in the province of Salamanca doubled between 2011 and 2021, with transdermal patches being the main form of presentation and, therefore, the prescription responsible for this increase. The trend in consumption is in line with national data for Spain, although it seems that the increase in the province of Salamanca has been even greater in recent years, probably influenced by a rapidly ageing population in this province. Despite this upward trend, the prevalence of fentanyl use in Spain is significantly lower than in the US, possibly reflecting differences in healthcare systems.

## Figures and Tables

**Table 1:** Statistical parameters obtained from regression analysis for total fentanyl use and split by route of administration. For those groups that showed a statistically significant trend, slopes were calculated, and a test for equality of slopes was performed. There is a positive and statistically significant upward trend for total fentanyl, and for the transdermal, oral, and sublingual routes of administration. The oral, sublingual, and nasal DHDs were summed to calculate the DHD for immediate-release fentanyl. Legend: n.s. not significant; *** *p*-value < 0.001.

Regression Analysis	Total	Transdermal	Oral	Sublingual	Nasal	Immediate-Release Fentanyl (3 Routes)
R^2^	0.9176	0.9430	0.3408	0.7863	0.002582	0.01976
Slope	0.1108 ± 0.012	0.1093 ± 0.008496		0.0032 ± 0.0005		
Difference between slopes (vs. Total fentanyl)		F = 0.0082 *p* = 0.9287 (n.s.) Pooled slope = 0.1087		F = 104.627 *p* < 0.0001 (***)		

**Table 2:** Statistical parameters obtained from regression analysis, for total fentanyl use and for the transdermal and oral routes. For those groups that showed a statistically significant trend, slopes were calculated, and a test for equality of slopes was performed. There is a positive and statistically significant upward trend for total fentanyl and for the transdermal route of administration. Legend: n.s. not significant.

Regression Analysis	Total	Transdermal	Oral
R^2^	0.9007	0.9313	0.3984
Slope	10.13 ± 0.9466	8.626 ± 0.9475	
Difference between slopes (vs. Total fentanyl)		Total vs. Transdermal F = 1.2671 *p* = 0.2751 (n.s.) Pooled slope = 9.3796	

**Table 3:** Statistical parameters obtained from regression analysis; slopes were calculated, and a test for equality of slopes was performed. There is a positive and statistically significant upward trend for total fentanyl use in both Salamanca and Spain. Legend: n.s. not significant.

Regression	Salamanca	Spain
R^2^	0.8535	0.9176
Slope	0.1081 ± 0.0124	0.0951 ± 0.0124
Difference between slopes	F = 0.65118 *p* = 0.4292 (n.s.) Pooled slope = 0.1016	

## Data Availability

The original contributions presented in the study are included in the article/Supplementary Materials. Further enquiries can be directed to the corresponding author/s.

## Supplementary Materials

The following supporting information can be downloaded at: https://www.mdpi.com/article/10.3390/healthcare12161619/s1: Figure S1. Year-on-year change in fentanyl consumption in the province of Salamanca between 2011 and 2022, for total DHDs, as well as for the two main routes of administration: transdermal and oral. Figure S2. Change in the use of fentanyl in the province of Salamanca and in Spain between 2011 and 2022, expressed as a percentage of change in the number of DHDs compared with 2011. Figure S3. Year-on-year variation in the consumption of fentanyl in the province of Salamanca and in Spain from 2011 to 2022.

## References

[1] Departamento de Medicamentos de Uso Humano, Ministerio de Sanidad, Servicios Sociales e Igualdad (MISAN) (2017). Informe de Utilización de Medicamentos. Utilización de Medicamentos Opioides en España Durante el Periodo 2008–2015. No. Informe U/OPI/V1/13022017. https://www.aemps.gob.es/medicamentosUsoHumano/observatorio/docs/opioides-2008-2015.pdf.

[2] Payen J.F., Chanques G., Mantz J., Hercule C., Auriant I., Leguillou J.L., Binhas M., Genty C., Rolland C., Bosson J.L. (2007). Current practices in sedation and analgesia for me-chanically ventilated critically ill patients: A prospective multicenter patient-based study. Anesthesiology.

[3] Aoki Y., Kato H., Fujimura N., Suzuki Y., Sakuraya M., Doi M. (2022). Effects of fentanyl administration in mechanically ventilated patients in the intensive care unit: A systematic review and meta-analysis. BMC Anesthesiol..

[4] Mehta S., Burry L., Fischer S., Martinez-Motta J.C., Hallett D., Bowman D., Wong C., Meade M.O., Stewart T.E., Cook D.J. (2006). Canadian survey of the use of sedatives, analgesics, and neuromuscular blocking agents in critically ill patients. Crit. Care Med..

[5] Daverio M., Von Borell F., Ramelet A.-S., Sperotto F., Pokorna P., Brenner S., Mondardini M.C., Tibboel D., Amigoni A., Ista E. (2022). Pain and sedation management and monitoring in pediatric intensive care units across Europe: An ESPNIC survey. Crit. Care..

[6] Koizumi T., Kurosawa H. (2020). Survey of analgesia and sedation in pediatric intensive care units in Japan. Pediatr. Int..

[7] Grape S., Schug S.A., Lauer S., Schug B.S. (2010). Formulations of fentanyl for the management of pain. Drugs.

[8] McWilliams K., Fallon M. (2013). Fast-acting fentanyl preparations and pain management. QJM.

[9] Stanley T.H. (2014). The fentanyl story. J. Pain.

[10] Brząkała J., Leppert W. (2019). The role of rapid onset fentanyl products in the management of breakthrough pain in cancer patients. Pharmacol. Rep..

[11] Comité Técnico Asesor del Dolor de Castilla y León (2017). Gerencia Regional de Salud Sanidad de Castilla y León, GRS SACYL. Criterios de uso de Fentanilo Liberación Rápida. SACYL. https://www.saludcastillayleon.es/sanidad/cm/facm/1359467/1050864-Folleto_Fentanilo%20de%20liberaci%C3%B3n%20r%C3%A1pida-Criterios%20de%20uso.pdf.

[12] Gardner E.A., McGrath S.A., Dowling D., Bai D. (2022). The Opioid Crisis: Prevalence and Markets of Opioids. Forensic Sci. Rev..

[13] Belzak L., Halvers J. (2018). The opioid crisis in Canada: A national perspective. Health Promot. Chronic Dis. Prev. Can..

[14] Shrestha S., Stopka T.J., Hughto J.M.W., Case P., Palacios W.R., Reilly B., Green T.C. (2021). Prevalence and correlates of non-fatal overdose among people who use drugs: Findings from rapid assessments in Massachusetts, 2017–2019. Harm Reduct. J..

[15] Biggar E., Papamihali K., Leclerc P., Hyshka E., Graham B., Taylor M., Payer D., Maloney-Hall B., Buxton J.A. (2021). Towards cross-Canada monitoring of the unregulated street drug supply. BMC Public Health.

[16] Green T.C., Park J.N., Gilbert M., McKenzie M., Struth E., Lucas R., Clarke W., Sherman S.G. (2020). An assessment of the limits of detection, sensitivity and specificity of three devices for public health-based drug checking of fentanyl in street-acquired samples. Int. J. Drug Policy.

[17] Martinez S., Jones J.D., Brandt L., Campbell A.N.C., Abbott R., Comer S.D. (2020). The Increasing Prevalence of Fentanyl: A Urinalysis-Based Study Among Individuals with Opioid Use Disorder in New York City. Am. J. Addict..

[18] Agencia Española de Medicamentos y Productos Sanitarios (AEMPS) (2018). Ministerio de Sanidad Servicios Sociales e Igualdad (MISAN)Fentanilo de Liberación Inmediata: Importancia de Respetar las Condiciones de uso Autorizadas. https://www.aemps.gob.es/informa/notasInformativas/medicamentosUsoHumano/seguridad/2018/docs/NI_MUH_FV-5_2018-Fentanilo.pdf.

[19] Arrieta Loitegui M., Caro Teller J.M., Rosas Espinoza C., Ferrari Piquero J.M. (2020). Comparación del consumo intrahospitalario de fentanilo de liberación inmediata en 2014 y 2017: ¿ uso o abuso?. Rev. Española de Salud Pública.

[20] Observatorio Español de las Drogas y las Adicciones (2023). Informe 2023. Alcohol, Tabaco y Drogas Ilegales en España.

[21] Observatorio Español de las Drogas y las Adicciones (2023). Estadísticas 2023. Alcohol, Tabaco y Drogas Ilegales en España.

[22] International Narcotics Control Board (INCB) (2023). Narcotic Drugs—Technical Report.

[23] Díaz Madero A., Ramos Pollo D., Martín González M. (2009). Evolución del consumo de opioides en Castilla y León desde el año 2000 al 2006. Med. Paliativa..

[24] Consejería de Sanidad de la Junta de Castilla y León (SACYL) Portal del Medicamento. https://www.saludcastillayleon.es/portalmedicamento/es/.

[25] Departamento de Medicamentos de Uso Humano Agencia Española de Medicamentos y Productos Sanitarios (AEMPS). Ministerio de Sanidad, Servicios Sociales e Igualdad (MISAN). Observatorio de uso de Medicamentos. Utilización de Medicamentos Opioides en España. https://www.aemps.gob.es/medicamentos-de-uso-humano/observatorio-de-uso-de-medicamentos/utilizacion-de-medicamentos-opioides-en-espana/.

[26] Substance Abuse and Mental Health Services Administration (2023). Center for Behavioral Health Statistics and Quality, Substance Abuse and Mental Health Services Administration. Key Substance Use and Mental Health Indicators in the United States: Results from the 2022 National Survey on Drug Use and Health (HHS Publication No. PEP23-07-01-006, NSDUH Series H-58). https://www.samhsa.gov/data/report/2022-nsduh-annual-national-report.

[27] Guastella V., Delorme J., Chenaf C., Authier N. (2022). The Prevalence of Off-label Prescribing of Transmucosal Immediate-Release Fentanyl in France. J. Pain Symptom Manag..

[28] Wirz S., Wiese C.H., Zimmermann M., Junker U., Heuser-Grannemann E., Schenk M. (2013). Rapid release fentanyl administration forms. Comments of the Working Group on Tumor Pain of the German Pain Society. Schmerz.

[29] Nunez-Olarte J.M., Alvarez-Jimenez P. (2011). Emerging opioid abuse in terminal cancer patients taking oral transmucosal fentanyl citrate for breakthrough pain. J. Pain Symptom Manag..

[30] EUDA (2024). Drug-Induced Deaths. European Drug Report.

[31] Salgueiro-Gonzalez N., Béen F., Bijlsma L., Boogaerts T., Covaci A., Baz-Lomba J.A., Kasprzyk-Hordern B., Matias J., Ort C., Bodík I. (2024). Influent wastewater analysis to investigate emerging trends of new psychoactive substances use in Europe. Water Res..

[32] Griffiths P.N., Seyler T., De Morais J.M., Mounteney J.E., Sedefov R.S. (2023). Opioid problems are changing in Europe with worrying signals that synthetic opioids may play a more significant role in the future. Addiction.

[33] Comisión Permanente de Farmacia (2021). Ministerio de Sanidad, Asuntos Sociales e Igualdad (MISAN). Plan de Optimiza-Ción de la Utilización de Analgésicos Opioides en dolor Crónico no Oncológico en el Sistema Nacional de Salud. https://www.sanidad.gob.es/areas/farmacia/publicaciones/planOptimizacion/docs/opioides/20210927_Plan_Optimizacion_Opioides.pdf.

[34] Butler S.F., Budman S.H., Fernandez K.C., Houle B., Benoit C., Katz N., Jamison R.N. (2007). Development and validation of the Current Opioid Misuse Measure. Pain.

[35] Butler S.F., Fernandez K., Benoit C., Budman S.H., Jamison R.N. (2008). Validation of the revised Screener and Opioid Assessment for Patients with Pain (SOAPP-R). J. Pain.

[36] Karamouzian M., Papamihali K., Graham B., Crabtree A., Mill C., Kuo M., Young S., Buxton J.A. (2020). Known fentanyl use among clients of harm reduction sites in British Columbia, Canada. Int. J. Drug Policy.

