# Supplementary figures and images for: Evolution of Fentanyl Prescription Patterns and Administration Routes in Primary Care in Salamanca, Spain: A Comprehensive Analysis from 2011 to 2022
Source: Healthcare (Basel). 2024 Aug 14;12(16):1619. doi: 10.3390/healthcare12161619 (PMC11353527; doi:10.3390/healthcare12161619)

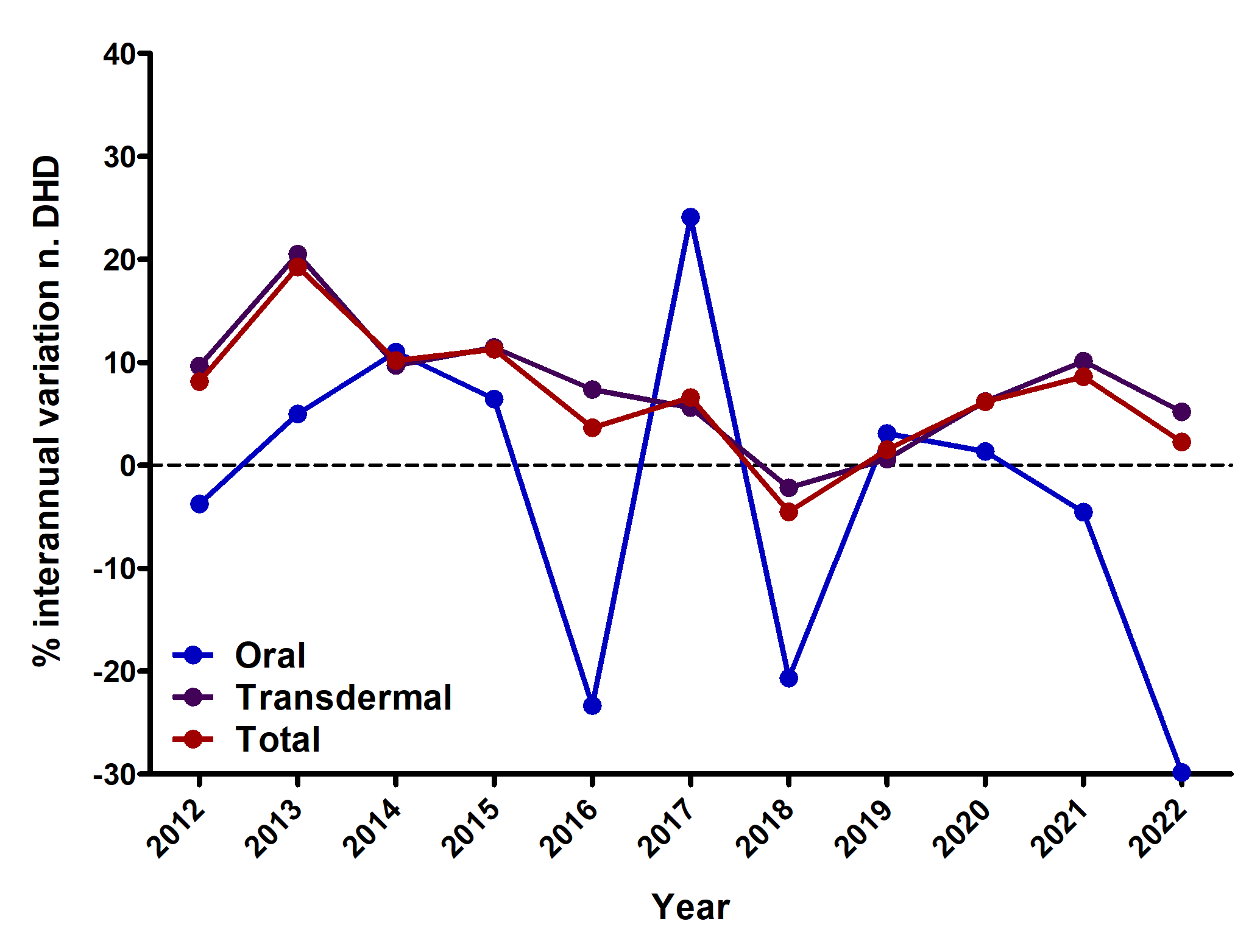

Supplement: Supplementary file 1 [file healthcare-12-01619-s001.zip › Figure_S1.tif]

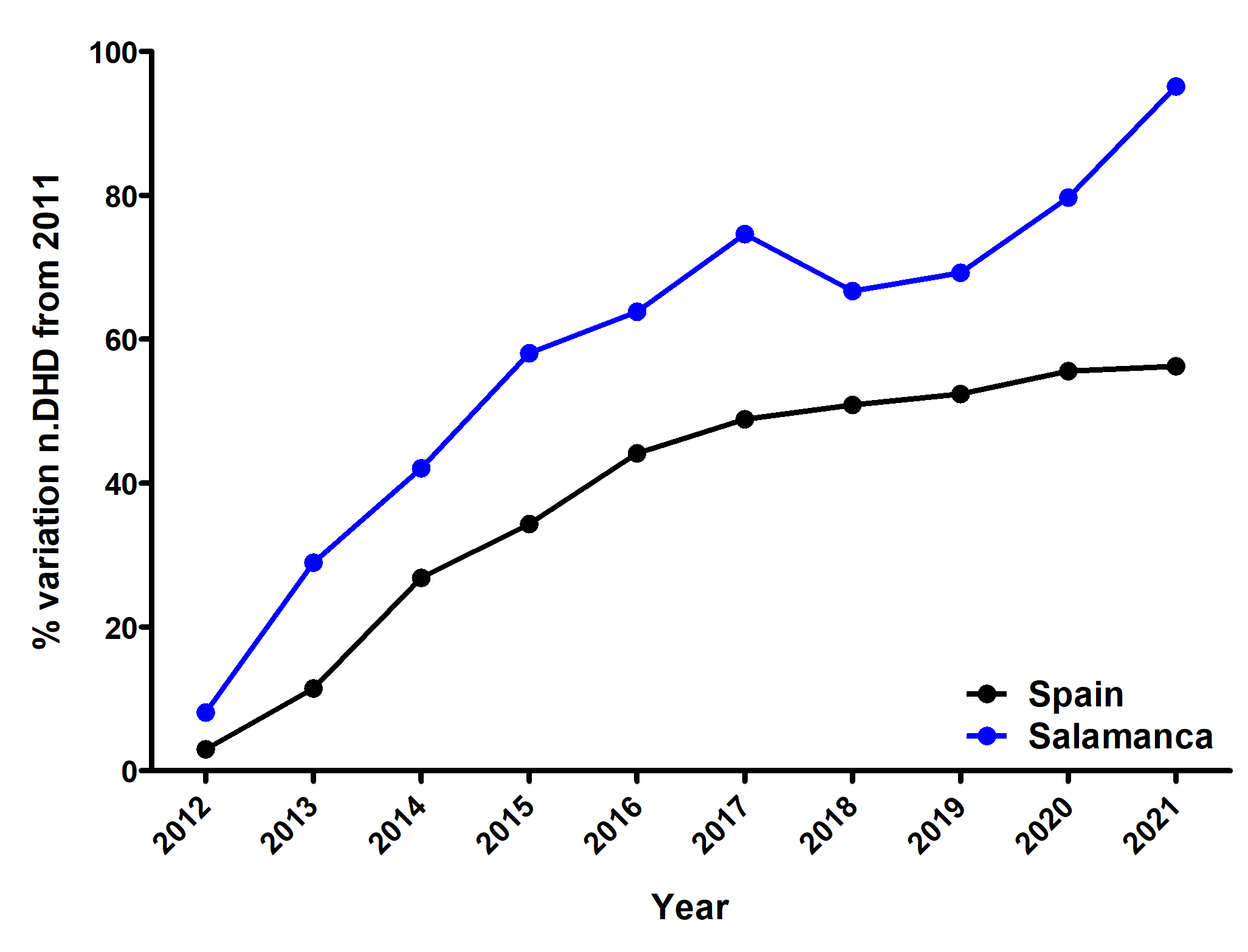

Supplement: Supplementary file 1 [file healthcare-12-01619-s001.zip › Figure_S2.tif]

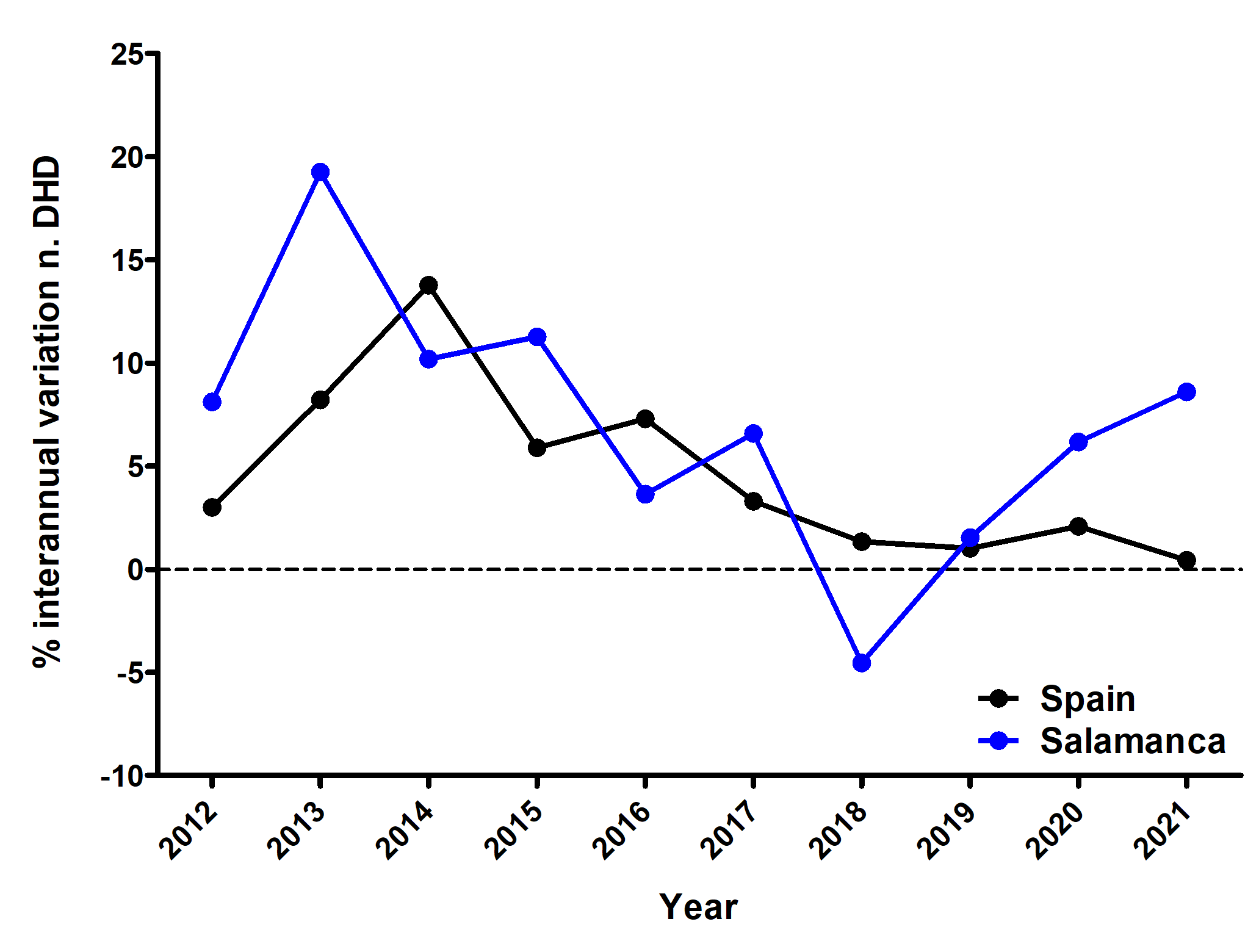

Supplement: Supplementary file 1 [file healthcare-12-01619-s001.zip › Figure_S3.tif]
